# Racial and ethnic differences in predictors of vitamin D among pregnant women in south-eastern USA

**DOI:** 10.1017/jns.2019.4

**Published:** 2019-02-28

**Authors:** Devika Chawla, Julie L. Daniels, Sara E. Benjamin-Neelon, Bernard F. Fuemmeler, Cathrine Hoyo, Jessie P. Buckley

**Affiliations:** 1 Department of Epidemiology, University of North Carolina Gillings School of Global Public Health, Chapel Hill, NC 27599-7400, USA; 2 Department of Health, Behavior and Society, Johns Hopkins University Bloomberg School of Public Health, Baltimore, MD 21205, USA; 3 Department of Health Behavior and Policy, Virginia Commonwealth University School of Medicine, Richmond, VA 23298, USA; 4 Department of Biological Sciences, North Carolina State University, Raleigh, NC 27695, USA; 5 Environmental Health and Engineering, Johns Hopkins University Bloomberg School of Public Health, Baltimore, MD 21205, USA

**Keywords:** Vitamin D, 25-Hydroxyvitamin D, Race, Ethnicity, 25(OH)D, 25-hydroxyvitamin D.

## Abstract

Insufficient vitamin D during pregnancy increases risk of adverse outcomes, with known differences by race/ethnicity. We sought to determine whether predictors of vitamin D insufficiency vary by race/ethnicity in an ethnically diverse pregnancy cohort. Plasma 25-hydroxyvitamin D concentrations and patient characteristics were measured at first prenatal visit to prenatal clinics in south-eastern USA between 2009 and 2011 (*n* 504). Prevalence ratios (PR) and 95 % CI were estimated using multivariable regression to quantify predictors of vitamin D insufficiency, overall and by race/ethnicity. In race/ethnicity-stratified models, season was most associated with vitamin D insufficiency among non-Hispanic white women; PR for winter *v.* summer were 3·58 (95 % CI 1·64, 7·81) for non-Hispanic white, 1·52 (95 % CI 1·18, 1·95) for Hispanic and 1·14 (95 % CI 0·99, 1·30) for non-Hispanic black women. Although women with darker skin tones are most vulnerable to prenatal vitamin D insufficiency, season may be more strongly associated with insufficiency among women with lighter skin tones.

Over half of pregnant women in the USA have suboptimal vitamin D levels, and non-Hispanic black women are disproportionally affected^(^^1^^,^^2^^)^. Recent epidemiological studies suggest that insufficient prenatal vitamin D increases risk of adverse pregnancy outcomes, including pre-eclampsia, pregnancy loss, preterm delivery and fetal growth restriction^(^^3^^–^^5^^)^. However, pregnancy-specific guidelines have been widely accepted and current guidelines for vitamin D sufficiency in the general population differ among authoritative bodies^(^^6^^)^.

Race and ethnicity are known to be strong predictors of vitamin D, as measured by 25-hydroxyvitamin D (25(OH)D) concentrations. Previous studies measuring 25(OH)D concentrations during pregnancy have reported higher concentrations in non-Hispanic white women compared with Hispanic and non-Hispanic black women^(^^7^^,^^8^^)^. Some studies suggest that the aetiological effects of vitamin D on health outcomes differ by race/ethnicity^(^^4^^,^^5^^,^^7^^)^. Moreover, a recent study reported that effects of prenatal 25(OH)D concentrations on birth outcomes were modified by race/ethnicity^(^^9^^)^.

Despite these known differences, few studies have assessed whether predictors of 25(OH)D concentrations also vary by race/ethnicity. Understanding the relative importance of vitamin D predictors in different race/ethnicity groups is important for (1) identifying populations at high risk of vitamin D deficiency during pregnancy, (2) identifying race/ethnicity-specific modifiable risk factors for vitamin D deficiency and (3) adequately modelling effect measure modification by race/ethnicity in aetiological studies of vitamin D.

To address this gap, we measured plasma concentrations of 25(OH)D, a biomarker of vitamin D exposure, in an ethnically diverse pregnancy cohort in south-eastern USA and examined how predictors of 25(OH)D concentrations vary by race/ethnicity.

## Methods

### Study population

The Newborn Epigenetic Study (NEST) followed a cohort of pregnant women who were seeking prenatal care from four healthcare facilities in a south-eastern US county from 2009 to 2011. Women were eligible to participate if they were 18 years of age or older, spoke English or Spanish, and planned to receive obstetric care at one of two facilities in Durham county (latitude: 36° N). Interviewers administered questionnaires to women at enrolment to ascertain information on sociodemographic characteristics and behaviours. Additional details on study design of the NEST are available elsewhere^(^^10^^)^. Plasma 25(OH)D concentrations were measured in the first 510 enrolled pregnant women, of whom 504 had valid measures and were included in this analysis. The NEST was approved by the Duke University Institutional Review Board and written consent was obtained from all women participating as study subjects.

### Vitamin D measurement

Participating mothers provided a non-fasting blood sample at the time of enrolment, typically during the 1st or 2nd trimester (median 11·3 (interquartile range 8·9–15·6) weeks of gestation). Plasma samples were aliquoted and stored at −80°C until analysis. We measured plasma concentrations of 25(OH)D using the commercially available immunodiagnostic system (IDS Fountain Hills AZ) enzyme immunoassay (catalogue no. AC57F1) (Craft Technologies, Inc.). We performed the immunoassay in duplicate using 25 μl plasma, according to the manufacturer's instructions, and % CV of all sample duplicates was <13 %. We categorised women as having sufficient (≥50 nmol/l) or insufficient (<50 nmol/l) vitamin D status (hereafter referred to as vitamin D sufficient or insufficient), according to the Institute of Medicine (now the National Academy of Medicine) guidelines^(^^11^^,^^12^^)^.

### Patient characteristics

We identified potential predictors of vitamin D from current literature. We assigned season of blood draw based on the date of the sample collection and categorised the season as winter (December–February), spring (March–May), summer (June–August) and autumn (September–November). We ascertained self-reported maternal characteristics, including pre-pregnancy weight, height, age, education, marital status, parity, prenatal vitamin use, multivitamin use and smoking, by questionnaire at enrolment. Women self-identified as non-Hispanic white, non-Hispanic black, or Hispanic. We calculated pre-pregnancy BMI in kg/m^2^ as self-reported pre-pregnancy weight/(height x height) and categorised women as underweight (<18·5 kg/m^2^), normal weight (18·5–24·9 kg/m^2^), overweight (25·0–29·9 kg/m^2^) or obese (≥30·0 kg/m^2^). We defined maternal smoking as any current smoking at time of enrolment. Similarly, we defined prenatal vitamin and (non-prenatal) multivitamins as any current use at time of enrolment. Gestational age at enrolment was derived from last menstrual period and categorised as 1st (<14 completed weeks of gestation) or 2nd trimester (14–27 completed weeks of gestation).

### Statistical analysis

We tabulated covariate distributions by vitamin D status (defined as insufficient or sufficient) and computed mean 25(OH)D concentrations by covariate groupings, overall and by race/ethnicity. 25(OH)D concentrations were normally distributed in our population. To evaluate independent predictors of low 25(OH)D concentrations in our study population, we estimated prevalence ratios and 95 % CI by simultaneously including all patient characteristics (defined above) in log-binomial regression models predicting prevalence of insufficient vitamin D status^(^^13^^)^. We also ran models stratified by race/ethnicity to examine predictors separately for non-Hispanic white, non-Hispanic black and Hispanic women (Table 3). To formally test whether predictors of 25(OH)D concentrations differed by race/ethnicity, we fit an equivalent model that included product terms between race/ethnicity and all predictors^(^^14^^)^. For main effects, we considered estimates to be statistically significant at *P* < 0·05. We considered there to be significant differences by race/ethnicity if the Wald type I *P* value for the product term was <0·1. In sensitivity analyses, we ran linear regression models examining predictors of continuous plasma 25(OH)D concentrations and found similar results (data not shown). We performed statistical analyses in SAS 9.3 (SAS Institute).

### Ethical standards disclosure

This study was conducted according to the guidelines laid down in the Declaration of Helsinki and all procedures involving human subjects/patients were approved by the Institutional Review Board of Duke University Medical Center. Written informed consent was obtained from all patients.

## Results and discussion

We report the distribution of participant characteristics in the overall population and among women with sufficient or insufficient vitamin D status in Table 1 and observed mean 25(OH)D concentrations, overall and by race/ethnicity, in Table 2. The study population was distributed across non-Hispanic white (*n* 144), non-Hispanic black (*n* 191) and Hispanic (*n* 169) women. The prevalence of vitamin D insufficiency was 47 % for non-Hispanic white women, 91 % for non-Hispanic black women and 75 % for Hispanic women. Non-Hispanic black women in winter months had the lowest observed 25(OH)D concentrations of any group (mean = 28·8 (sd = 9·4) nmol/l), while white women in summer months had the highest (mean = 58·3 (sd = 11·8) nmol/l). Mean concentrations of 25(OH)D were lower among obese compared with normal-weight women among all race/ethnicity groups. Women who used prenatal vitamins had similar observed 25(OH)D concentrations to those who did not (Table 2).

**Table 1.** Study population characteristics for women with overall, sufficient and insufficient vitamin D status* (Numbers of participants and percentages)

	Overall (*n* 504)		Sufficient (*n* 135)		Insufficient (*n* 369)	
	*n*	%	*n*	%	*n*	%
Season of blood draw						
Winter	113	23	14	10	99	27
Spring	157	31	28	21	129	35
Summer	140	28	58	43	82	22
Autumn	94	19	35	26	59	16
Race/ethnicity						
Non-Hispanic white	144	29	76	56	68	18
Non-Hispanic black	191	38	17	13	174	47
Hispanic	169	34	42	31	127	34
Pre-pregnancy BMI						
Underweight	17	3	7	5	10	3
Normal	191	38	70	52	121	33
Overweight	153	30	35	26	118	32
Obese	143	29	23	17	120	32
Maternal age at delivery (years)						
<25	172	34	29	21	143	39
25–29	137	27	33	24	104	28
30–34	121	24	49	36	72	20
≥35	74	15	24	18	50	14
Maternal education						
<High school graduate	169	33	29	21	140	38
High school graduate/GED	109	22	26	19	83	22
Some college	76	15	11	8	65	18
College graduate	147	29	67	50	80	22
Missing	3		2		1	
Marital status						
Never married	141	28	17	13	124	34
Married	191	38	81	60	110	30
Living with partner	138	28	32	24	106	29
Divorced/widowed/other	28	6	4	3	24	7
Missing	6		1		5	
Parity						
0	176	35	45	33	131	36
1+	328	65	90	67	238	65
Prenatal vitamin						
Yes	279	56	82	62	198	55
No	216	44	51	38	165	45
Missing	8		2		6	
Additional multivitamins						
Yes	17	3	9	7	8	2
No	474	97	122	93	352	98
Missing	13		4		9	
Smoking during pregnancy						
Yes	79	16	16	12	62	17
No	413	84	116	88	299	83
Missing	11		3		8	
Gestational age at enrolment						
1st trimester	312	62	90	67	222	60
2nd trimester	192	38	45	33	147	40

GED, general education development.

* Insufficient vitamin D status is defined as plasma concentrations <50 nmol/l, while sufficient vitamin D status is defined as plasma concentrations ≥50 nmol/l.

**Table 2.** Plasma 25-hydroxyvitamin D concentrations (nmol/l) in the study population, overall and by race/ethnicity (Mean values and standard deviations)

	Overall (*n* 504)		White (*n* 144)		Black (*n* 191)		Hispanic (*n* 169)	
	Mean	sd	Mean	sd	Mean	sd	Mean	sd
Overall	41·3	14·3	51·1	12·9	33·4	11·8	41·8	12·6
Season								
Winter	35·0	13·1	45·5	13·1	28·8	9·4	33·7	11·4
Spring	38·5	12·8	46·7	11·2	31·7	10·6	39·4	12·6
Summer	47·8	13·5	58·3	11·8	39·0	11·2	48·0	11·1
Autumn	43·8	14·7	53·3	11·8	35·2	14·2	43·4	9·8
Pre-pregnancy BMI								
Underweight	42·9	13·8	49·3	11·5	33·0	16·3	43·7	10·0
Normal	45·0	14·6	52·7	13·5	37·3	13·15	43·4	12·4
Overweight	40·1	14·3	51·6	13·6	30·8	9·8	41·6	13·1
Obese	37·3	12·8	47·4	10·9	31·9	11·0	39·3	12·1
Maternal age (years)								
<25	38·4	13·2	50·7	14·6	33·8	11·9	41·4	11·2
25–29	40·0	15·2	50·7	14·0	31·6	13·0	40·1	12·8
30–34	45·6	14·4	53·0	11·8	33·2	9·0	43·4	14·4
≥35	43·3	13·1	48·2	12·4	35·8	12·2	43·9	12·1
Maternal education								
<High school graduate	39·0	12·9	43·8	13·0	33·9	12·7	41·3	12·2
High school graduate/GED	39·5	15·2	57·4	12·7	33·9	12·6	40·3	13·3
Some college	36·1	12·2	44·7	11·8	32·1	10·8	41·4	10·5
College graduate	47·7	14·2	51·9	12·4	33·0	10·6	50·5	12·1
Marital status								
Never married	36·3	12·5	53·5	13·0	33·9	11·9	38·7	9·7
Married	46·6	14·2	51·3	12·6	32·4	10·1	43·8	13·7
Living with partner	40·2	14·1	50·6	15·6	32·6	12·4	42·3	12·7
Divorced/widowed/other	37·2	12·8	41·4	13·2	34·4	13·4	38·5	12·5
Parity								
0	42·1	13·2	50·1	11·9	35·3	12·2	41·3	9·4
1+	40·8	14·9	51·9	13·7	32·3	11·5	42·0	13·5
Prenatal vitamin								
Yes	43·0	14·2	51·1	13·0	34·2	12·3	43·6	12·3
No	39·0	14·3	51·0	13·0	32·3	11·3	39·3	12·9
Missing	45·1	7·5	*n *< 5		*n* < 5		*n* < 5	
Additional multivitamin								
Yes	47·8	16·3	60·5	8·6	35·4	14·0	52·4	3·3
No	41·0	14·2	50·6	13·0	33·1	11·6	41·5	12·6
Missing	43·9	13·0	*n* < 5		39·0	14·4	48·0	9·2
Smoking during pregnancy								
No	42·1	14·0	51·4	13·1	33·8	11·4	41·8	12·1
Yes	37·0	15·2	48·8	12·2	31·9	12·9	44·7	20·5
Missing	39·4	14·5	*n* < 5		37·8	12·2	38·4	18·1
Gestational age at enrolment (weeks)								
0–10	41·6	14·9	51·5	14·2	31·9	11·5	42·5	11·6
11–20	41·0	13·7	50·7	11·9	33·3	10·7	41·0	12·7
21–35	41·7	15·3	51·6	15·5	37·0	15·3	43·7	14·0

GED, general education development.

In adjusted models, season of blood draw (specifically winter) and race/ethnicity (all race/ethnicity groups) were the strongest predictors of vitamin D insufficiency in the overall study population (Table 3). Season of blood draw remained the strongest predictor in race-specific adjusted models, and was an especially strong predictor of vitamin D insufficiency for non-Hispanic white women. Higher pre-pregnancy BMI was significantly associated with higher prevalence of vitamin D insufficiency in the overall model, and in stratified models persisted as a significant predictor for non-Hispanic black women (Table 3). Lack of prenatal vitamin use was not a statistically significant predictor of vitamin D insufficiency in any racial/ethnic groups (Table 3). Lower maternal education was significantly associated with higher prevalence of vitamin D insufficiency in the overall models, and was especially strong for non-Hispanic white and Hispanic women (Table 3).

**Table 3.** Predictors of insufficient vitamin D status, overall and by race/ethnicity* (Prevalence ratios (PR) and 95 % confidence intervals)

	Overall		White		Black†		Hispanic‡	
	PR	95 % CI	PR	95 % CI	PR	95 % CI	PR	95 % CI
Season					*P* < 0·01§		*P* < 0·01§	
Winter	1·55	1·32, 1·81	3·58	1·64, 7·81	1·14	0·99, 1·30	1·52	1·18, 1·95
Spring	1·40	1·20, 1·63	3·56	1·63, 7·74	1·16	1·01, 1·32	1·28	1·00, 1·62
Summer	Ref		Ref		Ref		Ref	
Autumn	1·14	0·94, 1·40	1·72	0·69, 4·29	1·00	0·83, 1·20	1·36	1·02, 1·83
Race/ethnicity								
White	Ref		N/A		N/A		N/A	
Black	1·56	1·28, 1·90	N/A		N/A		N/A	
Hispanic	1·34	1·07, 1·69	N/A		N/A		N/A	
Pre-pregnancy BMI					*P* = 0·90		*P* = 0·99	
Normal	Ref		Ref		Ref		Ref	
Overweight	1·15	1·00, 1·31	1·05	0·69, 1·61	1·17	1·02, 1·34	1·02	0·84, 1·24
Obese	1·21	1·06, 1·38	1·33	0·86, 2·06	1·11	0·98, 1·26	1·12	0·87, 1·45
Maternal age (years)					*P* = 0·49		*P* = 0·59	
<25	0·96	0·79, 1·17	1·11	0·52, 2·36	1·11	0·94, 1·32	1·07	0·78, 1·46
25–29	1·02	0·84, 1·25	0·91	0·55, 1·51	1·05	0·90, 1·23	1·11	0·79, 1·56
30–34	0·96	0·77, 1·19	0·70	0·43, 1·16	1·11	0·96, 1·30	1·05	0·76, 1·44
≥35	Ref		Ref		Ref		Ref	
Parity					*P* = 0·02§		*P* = 0·18§	
0	Ref		Ref		Ref		Ref	
1+	0·89	0·78, 1·00	0·70	0·47, 1·04	1·05	0·94, 1·19	0·91	0·73, 1·12
Vitamin use‖					*P* = 0·93§		*P* = 0·56§	
Yes	0·94	0·62, 1·43	1·20	0·79, 1·84	1·06	0·97, 1·16	0·99	0·82, 1·20
No	Ref		Ref		Ref		Ref	
Smoking during pregnancy					*P* = 0·23		*P* = 0·15	
Yes	0·99	0·87, 1·13	1·41	0·85, 2·33	1·00	0·89, 1·12	0·71	0·36, 1·42
No	Ref		Ref		Ref		Ref	
Maternal education					*P* = 0·15		*P* = 0·58	
<High school graduate	1·30	1·03, 1·65	2·07	1·03, 4·14	0·93	0·78, 1·11	2·62	1·10, 6·25
High school graduate/GED	1·11	0·89, 1·39	2·07	1·03, 4·14	0·90	0·77, 1·05	2·50	1·03, 6·09
Some college	1·23	1·01, 1·50	1·40	0·76, 2·57	1·00	0·89, 1·12	2·56	0·99, 6·59
College graduate	Ref		Ref		Ref		Ref	
Marital status					*P* = 0·05§		*P* = 0·05§	
Never married	1·10	0·94, 1·29	0·95	0·42, 2·15	1·01	0·90, 1·13	1·27	0·99, 1·62
Married	Ref		Ref		Ref		Ref	
Living with partner	1·00	0·85, 1·18	0·46	0·24, 0·91	0·90	0·77, 1·06	1·10	0·85, 1·42
Divorced/widowed/other	1·00	0·78, 1·24	0·72	0·27, 1·92	0·97	0·80, 1·18	1·07	0·75, 1·54
Gestational age at enrolment					*P* = 0·42		*P* = 0·46	
1st trimester	Ref		Ref		Ref		Ref	
2nd trimester	1·03	0·92, 1·15	1·18	0·80, 1·73	0·98	0·89, 1·09	0·98	0·82, 1·16

Ref, reference; N/A, not applicable; GED, general education development.

* Insufficient vitamin D status is defined as plasma concentrations <50 nmol/l.

† *P* values for effect-measure modification comparing non-Hispanic black with non-Hispanic white women.

‡ *P* values for effect-measure modification comparing Hispanic with non-Hispanic white women.

§ Statistically significant effect-measure modification (*P* < 0·10).

‖ Includes any use of prenatal vitamin or multivitamins at time of enrolment.

In models stratified by race/ethnicity, we observed some statistically significant differences in predictors of vitamin D insufficiency. Relationships of season and marital status with vitamin D insufficiency were significantly different among white women as compared with non-Hispanic black (*P* < 0·01 for season, *P* = 0·05 for marital status) or Hispanic women (*P* < 0·01 for season, *P* = 0·05 for marital status). In addition, the relationship between parity and vitamin D insufficiency was significantly different among non-Hispanic white women as compared with among non-Hispanic black women (*P* = 0·02), but not as compared with Hispanic women (*P* = 0·18).

We identified several predictors of vitamin D insufficiency in pregnant women that differed according to race/ethnicity, including season of blood draw, parity and marital status. While season and race/ethnicity are known to be independently associated with vitamin D status, our study is the first to quantify differences in seasonal patterns of vitamin D insufficiency in some but not all race/ethnic groups in a multi-ethnic cohort of pregnant women.

Our results are consistent with findings from a recent study of three cohorts in the USA showing that predicted seasonal variations in prenatal 25(OH)D concentrations were stronger for white women as compared with black women^(^^8^^)^, but additionally compares seasonal patterns among Hispanic women. In our sample, seasonal variation for Hispanic women was less pronounced than among non-Hispanic white women but slightly stronger than among non-Hispanic black women. These differences in seasonal patterns persisted after adjusting for socio-economic status and other important predictors, suggesting that differences may be biologically driven. Darker skin tones require more UVB sunlight to generate previtamin D, which subsequently results in 25(OH)D in the body. This may, in part, explain why non-Hispanic black women have higher rates of vitamin D insufficiency than other racial/ethnic groups. Moreover, women with darker skin tones may be less sensitive to seasonal variations in sun exposure, since melatonin in darker skin tones reduces the efficiency of UVB absorption^(^^15^^)^. This may result in more dramatic seasonal patterns of 25(OH)D (e.g. higher peaks, lower nadirs) among non-Hispanic white women as compared with black and Hispanic women, which we observed in our study. Although we lacked information on skin tone, country of origin and detailed Hispanic ethnicity, our finding suggests that seasonal patterns of prenatal vitamin D differ by ethnicity as well as by race.

While associations between insufficient vitamin D status and parity and marital status differed significantly across race/ethnicity, these variables were not significant predictors of vitamin D insufficiency in any group and sample size was limited in stratified models. Being married was associated with slightly lower prevalence of insufficient vitamin D status for Hispanic women, but not for non-Hispanic black or non-Hispanic white women. This is probably explained by unmeasured confounding from broader socio-economic factors that are associated with marital status and may differ by race/ethnicity. Broadly, these results suggest that some – but not all – predictors of prenatal vitamin D may differ by race/ethnicity.

In overall models, season and race/ethnicity were strong predictors of vitamin D insufficiency during pregnancy as has been observed in other studies^(^^8^^)^. Pre-pregnancy BMI was a significant predictor of prenatal vitamin D insufficiency in the adjusted model, with overweight and obese women having an increased prevalence of vitamin D insufficiency. These results contribute to the small but growing literature that increased BMI may be associated with lower 25(OH)D concentrations^(^^16^^)^. Prenatal vitamin use and multivitamin use were not significant predictors of insufficiency. Most prenatal vitamins include vitamin D, but dosage varies by brand and adequacy of the dosing for vitamin D supplementation has been called into question^(^^17^^)^.

Understanding racial/ethnic differences in overall risk and predictors of vitamin D insufficiency has clinical implications for identifying women at high risk for vitamin D insufficiency during pregnancy. Findings from our study and others suggest that non-Hispanic black women are at higher risk of insufficiency and should be considered for targeted screenings and interventions. Moreover, while all women have some degree of seasonal variation in vitamin D, low sunlight exposure in the winter may have a greater impact on insufficiency among non-Hispanic white women. This racial/ethnic difference in seasonal patterns could be useful for informing when and how frequently women should be screened throughout the year. Future research on how patterns and predictors of vitamin D differ by race/ethnicity will help inform targeted screening and interventions for insufficient vitamin D during pregnancy. Another important implication of these findings is that future studies of race/ethnicity differences in associations between prenatal vitamin D and health outcomes should address the potential for race/ethnicity-specific confounding using appropriate statistical approaches^(^^14^^)^.

Our results should be considered in the context of some limitations. Most notably, 25(OH)D concentrations were measured once during pregnancy. To assess individual-level seasonal patterns, future studies should measure 25(OH)D at multiple time points during pregnancy. While we used a 25(OH)D enzyme immunoassay that has been validated in pregnant women, it is no longer considered the ‘gold-standard’ measure of 25(OH)D. However, validation studies have found concordance between immunoassays and the ‘gold-standard’ liquid chromatography measure of 25(OH)D^(^^18^^)^. Additionally, our enzyme immunoassay does not capture epimeric vitamin D metabolites, the biological relevance of which is currently debated in the field^(^^19^^)^. Because the vitamin D plasma collection and assay process was standardised within this study, we do not expect slight differences in 25(OH)D measurement to meaningfully change the interpretation of predictors and effect-measure modification by race/ethnicity in our paper. We also lacked data on important predictors of vitamin D such as amount of sun exposure and intake of vitamin D-rich foods. Future studies should replicate these results in geographically diverse study populations and using more detailed covariate data.

## Conclusions

In this multi-ethnic population of pregnant women in south-eastern USA, we confirmed that non-Hispanic black women are most at risk for vitamin D insufficiency. Additionally, we found evidence that certain predictors of vitamin D insufficiency during pregnancy – most notably, season – were statistically different by race/ethnicity with greater influence on insufficiency among non-Hispanic white women. Future studies identifying pregnancies at risk of vitamin D insufficiency should consider how predictors interact with race/ethnicity.
